# Sortal anaphora resolution to enhance relation extraction from biomedical literature

**DOI:** 10.1186/s12859-016-1009-6

**Published:** 2016-04-14

**Authors:** Halil Kilicoglu, Graciela Rosemblat, Marcelo Fiszman, Thomas C. Rindflesch

**Affiliations:** Lister Hill National Center for Biomedical Communications, U.S. National Library of Medicine, 8600 Rockville Pike, Bethesda, 20894 MD USA

**Keywords:** Natural language processing, Sortal anaphora resolution, Biomedical literature, Semantic relation extraction

## Abstract

**Background:**

Entity coreference is common in biomedical literature and it can affect text understanding systems that rely on accurate identification of named entities, such as relation extraction and automatic summarization. Coreference resolution is a foundational yet challenging natural language processing task which, if performed successfully, is likely to enhance such systems significantly. In this paper, we propose a semantically oriented, rule-based method to resolve sortal anaphora, a specific type of coreference that forms the majority of coreference instances in biomedical literature. The method addresses all entity types and relies on linguistic components of SemRep, a broad-coverage biomedical relation extraction system. It has been incorporated into SemRep, extending its core semantic interpretation capability from sentence level to discourse level.

**Results:**

We evaluated our sortal anaphora resolution method in several ways. The first evaluation specifically focused on sortal anaphora relations. Our methodology achieved a F_1_ score of 59.6 on the test portion of a manually annotated corpus of 320 Medline abstracts, a 4-fold improvement over the baseline method. Investigating the impact of sortal anaphora resolution on relation extraction, we found that the overall effect was positive, with 50 % of the changes involving uninformative relations being replaced by more specific and informative ones, while 35 % of the changes had no effect, and only 15 % were negative. We estimate that anaphora resolution results in changes in about 1.5 % of approximately 82 million semantic relations extracted from the entire PubMed.

**Conclusions:**

Our results demonstrate that a heavily semantic approach to sortal anaphora resolution is largely effective for biomedical literature. Our evaluation and error analysis highlight some areas for further improvements, such as coordination processing and intra-sentential antecedent selection.

**Electronic supplementary material:**

The online version of this article (doi:10.1186/s12859-016-1009-6) contains supplementary material, which is available to authorized users.

## Background

Coreference can be defined as the relation between textual mentions that refer to the same real-world entity [1]. Coreference resolution is the natural language processing (NLP) task that is concerned with identifying such mentions and linking them to form coreference chains (clusters). While a key task in natural language understanding, coreference resolution remains far from being solved. Without reliable coreference resolution, NLP systems focusing on advanced semantic tasks such as relation extraction, automatic summarization, and question answering are likely to suffer. Consider the fragments of a MEDLINE abstract (PMID 21349396) in Example (1). The first two sentences begin the abstract and the third sentence concludes it.

1.
  *Pulmonary arterial hypertension (PAH) is a rare and progressive disease of the pulmonary arterial circulation ….*
  *There are currently *3 classes of drugs approvedfor the treatment of PAH:prostacyclinanalogues, endothelin receptor antagonists, andphosphodiesterase type 5 inhibitors. …
  *Although definitive evidence will require randomized and properly controlled long-term trials, the current evidence supports the long-term use of *these drugs* for the treatment of patients with PAH.*

The underlined mentions form a coreference cluster: *{3 classes of drugs approved for the treatment of PAH, {prostacyclin analogues, endothelin receptor antagonists, and phosphodiesterase type 5 inhibitors}, these drugs}*. Pairwise relation representation (such as *{{prostacyclin analogues, endothelin receptor antagonists, and phosphodiesterase type 5 inhibitors}, these drugs}*) can also be used to represent coreference. In the absence of coreference resolution, a relation extraction system could extract the following, not very informative, relation from the concluding sentence in Example (1) above:

- Drugs-TREATS-PAH

On the other hand, with successful coreference resolution, the system would be able to extract the following relations, which are more specific and informative.

- Prostacyclin analogues-TREATS-PAH
- Endothelin receptor antagonists-TREATS-PAH
- Phosphodiesterase type 5 inhibitors-TREATS-PAH

In doing so, the system would also be able to move beyond sentence level processing to discourse level processing, bringing us closer to discourse understanding, the ultimate goal in NLP.

Several types of coreference are often distinguished. For example, *anaphora* is a coreference relation in which a coreferential mention (*anaphor*), such as *these drugs* above, refers to a previously mentioned entity (*antecedent*) in text. *Cataphora* refers to a relation in which the coreferential expression (*cataphor*) refers to an entity subsequent to the expression in text (*consequent*). Broader views of coreference also consider relation types such as *bridging* and *appositive*. Different types of coreference can be indicated with mentions of varying types. For example, a major type of anaphora (*pronominal anaphora*) is indicated by pronouns, such as *it, their, itself*. In Example (1), the anaphor *these drugs* is a demonstrative noun phrase, therefore the anaphora relation can be referred to as *nominal anaphora*. Nominal anaphora is sometimes also referred to as *sortal anaphora* since such anaphors carry semantic type (sort) information, in contrast to pronominal expressions. For instance, in Example (1), the antecedents of *these drugs* can only be drug or drug class instances. In the studies focusing on coreference resolution in biomedical literature, sortal anaphors have attracted most attention, since they occur more frequently than other types. Castaño and Pustejovsky [2] found that approximately 60 % of anaphora instances in their corpus of MEDLINE abstracts were sortal. This was confirmed by Gasperin and Briscoe [3], who found that the majority of anaphora instances involved definite and demonstrative noun phrases in their corpus of full-text articles about *Drosophila melanogaster*.

### SemRep semantic interpreter

SemRep [4] is a natural language processing tool that extracts semantic relations, also referred to as predications, from biomedical literature. Each predication is a logical subject-predicate-logical object triple, whose elements are drawn from the UMLS knowledge sources [5]; the subject and object are concepts from the UMLS Metathesaurus and the predicate is a relation type from an expanded version of the UMLS Semantic Network. SemRep extracts a wide range of predicates regarding clinical medicine (e.g., TREATS, DIAGNOSES, ADMINISTERED_TO), substance interactions (e.g., STIMULATES, INHIBITS), genetic basis of disease (e.g., CAUSES, PREDISPOSES), and pharmacogenomics (e.g., AUGMENTS, DISRUPTS). From the input sentence in Example (2a), SemRep generates the three predications in Example (2b). Mentions corresponding to the predication arguments are underlined and those corresponding to the predicates are in bold.

(2)
  *The *antiviral agentamantadine* has been used to ****manage***Parkinson’s disease* or *levodopa-induced dyskinesias* for nearly 5 decades.*
  Amantadine-ISA-Antiviral Agents
    Amantadine-TREATS-Parkinson Disease
    Amantadine-TREATS-Levodopa-induced dyskinesias

SemRep processing relies on the UMLS SPECIALIST Lexicon [6], MedPost part-of-speech tagger [7], and underspecified syntactic analysis, and it is supported by MetaMap [8] for normalizing noun phrases to UMLS Metathesaurus concepts. Entrez Gene [9] serves as a supplementary source to the UMLS Metathesaurus with respect to gene/protein terms. Indicator rules are used to map lexical and syntactic phenomena to predicates. Indicators include lexical categories, such as verbs, nominalizations, and prepositions, and syntactic constructions, such as appositives or modifier-head structure in the simple noun phrase. For instance, in Example (2), the ISA predicate is indicated by the fact that its arguments are in a restrictive appositive construction ([*antiviral agent* ][*amantadine*]), while TREATS is lexically indicated by the verb *manage*. Using an ontology engineering approach [10], SemRep has been extended to domains that are outside the scope of the UMLS, such as disaster information management and public health. It has also been the basis for the Semantic MEDLINE web application [11] and SemMedDB, a PubMed-scale repository of semantic predications [12].

### Overview

In the current study, our goal has been to extend semantic interpretation capabilities of SemRep through anaphora resolution. Based on the observed prominence of sortal anaphora in biomedical literature [2, 3], we focused specifically on sortal anaphora resolution. Our rule-based methodology has a linguistic orientation and makes heavy use of UMLS semantic knowledge. A major contribution of our work is that our approach is not restricted to certain entity types, in contrast to other biomedical coreference resolution studies that focus on certain types of biomedical entities (e.g., chemicals, genes, cells [2], gene/proteins [13, 14]). Our study is also distinct in its focus on the impact of anaphora resolution on relation extraction at a large scale.

To refine and evaluate our approach, we annotated a set of 320 MEDLINE citations (titles and abstracts) for sortal anaphora. We evaluated our approach in several ways:

- Evaluation of sortal anaphora resolution on our annotated corpus
- Partial evaluation of sortal anaphora resolution on the Protein Coreference Dataset used in the BioNLP 2011 shared task [15]
- Evaluation of the impact of anaphora resolution on SemRep predications, for which we compared SemRep results with and without anaphora resolution on a separate set of 300 sentences
- Estimation of the quantitative effect of anaphora resolution at a larger scale, for which we compared the number of predications and relation types extracted by SemRep using anaphora resolution with that extracted without anaphora resolution on 1 million MEDLINE citations

The results show that our semantic approach is effective in recognizing sortal anaphora relations and that its incorporation into SemRep allows it to replace generic and uninformative relations with more specific and informative ones. We have incorporated our resolution approach into SemRep, making it an option in semantic processing. The annotated corpus of MEDLINE citations is available at http://skr3.nlm.nih.gov/SortalAnaphora/.

## Related work

Pioneering work in coreference resolution in general English focused on the interaction of pronominal anaphora with syntactic structure and discourse constraints [16–18]. Availability of corpora annotated for coreference, such as MUC7 [19], led to the prominence of supervised learning approaches for this task [20–22]. More recently, Haghighi and Klein [23] presented a deterministic algorithm that relies on syntactic, semantic, and discourse constraints and demonstrated good performance on several corpora. Lee et al. [24] extended this approach to propose a *sieve* architecture, which applies a set of deterministic coreference models (i.e., sieves) one at a time from highest to lowest precision, each sieve using the output of the previous one. Sieves include various string matching algorithms as well as speaker identification and pronoun resolution models. Their approach yielded state-of-the-art performance on the OntoNotes corpus [25], the current standard for evaluating coreference resolution systems for general English. The sieve architecture has been made part of the Stanford CoreNLP toolkit [9] and has been extended for multilingual coreference resolution by systems participating in the CoNLL 2012 Shared Task [26]. In addition to such end-to-end coreference resolution approaches, much effort has also been devoted to specific coreference resolution subtasks, such as recognizing non-referential mentions (e.g., pleonastic *it*) [27, 28] and anaphoricity detection (i.e., determining whether a mention is anaphoric or not) [29, 30].

In the biomedical domain, most coreference resolution research has involved biomedical literature. Castaño and Pustejovsky [2] focused on pronominal and sortal anaphora resolution of bio-entities, using semantic information from UMLS. They achieved 73.8 % F_1_ score on a small set of MEDLINE abstracts. Their algorithm is based on scoring potential antecedents according to their compatibility with the anaphor (e.g., number agreement). The candidate with the highest score is taken as the antecedent. A similar approach is taken by Kim et al. [13], who additionally investigated the role of Centering Theory [18], syntactic parallelism between the anaphor and the antecedent, coordinate noun phrases, and appositive constructions. Their approach yielded a F_1_ score of 63 % on a different set of MEDLINE abstracts. Yang et al. [31] and Torii and Vijay-Shanker [32], on the other hand, used supervised machine learning techniques for anaphora resolution. Taking a noun phrase clustering approach and casting the problem as a binary classification task, Yang et al. [31] achieved an F_1_ score of 81.7 % on a small set of MEDLINE abstracts. Focusing on sortal anaphora only, Torii and Vijay-Shanker [32] reported 71.6 % precision and 77 % recall in cross-validation experiments. Diverging from this line of research that focused on anaphora resolution in MEDLINE abstracts, Gasperin and Briscoe [3] annotated a corpus of five full-text molecular biology articles with sortal anaphora as well as with types of domain-specific associative coreference, such as homology and related biotype (e.g., the relationship between a gene and its product, a protein). Their annotation also included set-membership relations. The Bayesian probabilistic model they used achieved an F_1_ score of 57 % for coreference, although it performed poorly on associative relations. In line with the observation that coreference resolution could improve event extraction pipelines, a supporting task was proposed in the BioNLP 2011 shared task on biological event extraction [15]. A corpus of MEDLINE citations annotated for sortal and pronominal anaphora were provided to participants (BioNLP Protein Coreference Dataset). The best system was an adaptation of an existing coreference resolution system for newswire text and achieved an F_1_ score of 34 % [33], a significant performance loss from its performance on news text. Using Stanford CoreNLP sieve-based coreference resolution [24], Choi et al. [34] also obtained very poor results, confirming a trend of performance degradation of systems developed for the general domain. Underutilization of semantic information seems to be a factor for this trend [34]. Conversely, using domain-specific semantic information, Nguyen et al. [14] achieved an F_1_ score of 62.4 % on the same corpus. With a hybrid approach, D’Souza and Ng [35] reported an F_1_ score of 67.4 %. Improvements of varying degrees in event/relation extraction have been reported with the incorporation of coreference resolution [36–39]. A common feature of all these studies is that they focus on a pairwise relation representation of coreference and on specific entity types. In contrast, in the CRAFT corpus [40], full coreference chains are annotated in the spirit of the OntoNotes corpus, and all semantic types are considered (drugs, diseases, etc.). In addition, full-text articles are annotated, rather than abstracts. We are not aware of any resolution studies based on this recent corpus.

In the biomedical domain, coreference resolution has also been addressed in clinical narratives, drug labels, and consumer health texts. The 2011 i2b2/VA shared task was concerned with coreference resolution in clinical reports [41]. Training and evalution corpora annotated with coreference mention clusters were provided. Entity types considered for coreference included *problem*, *person*, *test*, *treatment*, and *anatomical site*. Rule-based, supervised learning, and hybrid approaches were proposed; a supervised learning approach which incorporated world knowledge and document structure [42] obtained the best results in one corpus, while a rule-based system [43] performed best in the other. Segura-Bedmar et al. [44] developed a corpus of drug interaction documents annotated with anaphora (DrugNer-AR) and obtained an F_1_ score of 76 % using Centering Theory constraints, semantic knowledge obtained with MetaMap [45], and drug class information for resolution. Névéol and Lu [46] improved the specificity of SemRep predications by using simple anaphora resolution heuristics in consumer medication texts and MeSH scope notes. Focusing on consumer health questions, Kilicoglu et al. [47] incorporated resolution of anaphora and ellipsis (a specific type of coreference characterized by the absence of one of the referents) to their question frame extraction pipeline and reported an 18 point improvement in F_1_ score in this task thanks to anaphora resolution.

## Methods

In this section, we first discuss our data and the annotation study. Next, we describe the algorithm that we developed for anaphora resolution. We conclude the section by describing our evaluation of the algorithm.

### Data and annotation

In order to develop, refine, and evaluate a sortal anaphora resolution module, we annotated a corpus of 320 MEDLINE citations with pairwise anaphora relations. Since we aimed at a general approach that takes into account all semantic types and consequently supports SemRep, we collected MEDLINE abstracts on a range of topics, including molecular biology and clinical medicine. Most molecular biology citations were previously used for evaluating some specific aspect of SemRep, such as nominalization processing [48], or were annotated for SemRep benchmarking [49]. Citations on clinical medicine were identified by issuing a SemMedDB query for the predicate types TREATS/PREVENTS and PROCESS_OF in the period from 2011 to 2013 and retrieving a random subset of the query results.

One hundred forty-nine citations were double-annotated by two of the authors of this paper (GR, MF) to develop and refine annotation guidelines as well as to calculate inter-annotator agreement. Once a satisfactory inter-annotator agreement was achieved, the rest of the corpus (171 citations) was annotated by one of the authors only (GR). We used the double-annotated portion of the corpus for training and refining the algorithm, and the other portion for testing. The corpus was pre-annotated with entities extracted by SemRep (using the default UMLS 2006AA release) to assist the annotators and simplify the task. Annotators were instructed to annotate the named entities missed by SemRep, if relevant for the sortal anaphora annotation task; however, we did not require them to annotate a specific semantic type for these entities and simply used SPAN as a generic type. For example, from the phrase *The flamenco gene*, SemRep only extracts a concept for the phrase head *gene* (the extracted concept is Genes), as there is no specific concept for *flamenco gene* in the UMLS. This is clearly an inadequate mapping for the phrase. Therefore, the full phrase was annotated as SPAN since it acts as the antecedent in an anaphora relation.

**Fig. 1:**
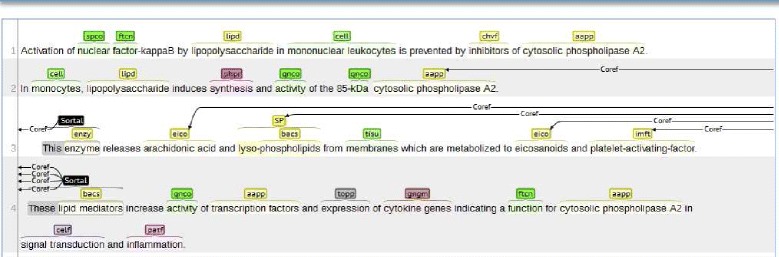
A sample annotation. Anaphora annotation in *brat* interface (PMID 10225377)

The annotation task consisted of two steps: a) identifying the anaphoric mentions in text and b) linking them to their antecedent(s). Some basic definitions and annotation guidelines were provided to the annotators, and these were refined in the course of the annotation study based on feedback and questions from the annotators. The annotation guidelines are provided as Additional file 1. The *brat* annotation tool [50] was used for the annotation task. A sample sortal anaphora annotation is provided in Fig. 1. The anaphoric mentions are abbreviated as *Sortal* and the links between the anaphoric mentions and the antecedents are abbreviated as *Coref*.

In the first phase of annotation, each annotator annotated 5 abstracts to familiarize themselves with the task. They discussed their annotations with the primary author, who adjudicated their differences. In the next step, each annotator independently annotated batches of approximately 50 abstracts at a time. After each batch, we calculated inter-annotator agreement to assess their progress, and the annotators reconciled their differences to create the gold standard reference for the batch. We calculated inter-annotator agreement for both the anaphoric mentions and the anaphora relations. As the inter-annotator agreement measure, we used the F_1_ score of one set of the annotations, with the other set taken as the gold standard, a measure often used for inter-annotator agreement in biomedical relation annotation [49, 51]. It has been shown that *κ* statistic [52], more typically used to calculate inter-annotator agreement, approximates F_1_ score in cases that lack a well-defined number of negative instances, which makes chance agreement close to zero [53]. After a satisfactory inter-annotator agreement was reached, one of the annotators (GR) annotated the rest of the corpus (171 citations) on her own.

### Algorithm

**Fig. 2:**
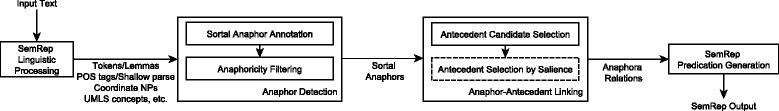
The sortal anaphora resolution pipeline. The high-level view of the sortal anaphora resolution pipeline and and its incorporation into SemRep

The anaphora resolution pipeline developed using the training set annotations is illustrated in Fig. 2.

The algorithm consists of two main phases: *anaphor detection* and *anaphor-antecedent linking*. The first phase is concerned with recognizing the noun phrases that are sortal anaphors and marking them as such. The second phase of the algorithm inspects these sortal anaphors and attempts to link them to their corresponding antecedents. Both phases of the algorithm presuppose a variety of linguistic information (lexical, morphological, syntactic, and semantic), made available by the core machinery of SemRep. Lexical and morphological information include individual tokens, their lemmas, part-of-speech tags, and inflection status (i.e., whether singular or plural) provided, to a large extent, by the SPECIALIST Lexicon [6]. Syntactic information includes noun phrases as well as their heads and modifiers, identified with a shallow syntactic parser. Some syntactic constructions, such as appositives and coordinate noun phrases, are relevant to anaphora resolution and are identified as well. Semantic information is provided by MetaMap and includes mappings from noun phrases to UMLS Metathesaurus concepts with their CUIs and semantic types. The algorithm also relies on taxonomic relations encoded in the UMLS Metathesaurus, such as the one between *Amantadine* and *Antiviral Agents*. Such relations are already extracted as part of SemRep’s hypernymy processing (i.e., ISA relations) [4]. Anaphora resolution requires that the entire previous discourse, not just the sentence with the anaphor, be available to identify antecedents. To facilitate this, we extended SemRep to take into account all linguistic information from previous sentences in addition to the current sentence, taking the first step toward discourse-level processing.

#### Anaphor detection

Anaphor detection is performed after the noun phrases in the text are mapped to UMLS Metathesaurus concepts by MetaMap. To recognize anaphors, we first identify noun phrases with particular determiners and adjectives that are used in sortal expressions. These include the definite article (*the*), demonstrative determiners (*this, that, these, those*), distributive determiners (*both, each, either, neither*) and a demonstrative adjective (*such*). The next steps are concerned with *anaphoricity* and filter out the noun phrases that are unlikely to be anaphors, based on morpho-syntactic features. Such noun phrases satisfy one of the following conditions:

- The noun phrase is in an appositive construction with the noun phrase that immediately follows it. For example, the definite noun phrase *the gene* in *…the gene, BRCA1, …* is not anaphoric.
- The noun phrase has a modifier that is mapped to the UMLS separately from the head; in other words, the modifier is an example of a *rigid designator* [2]. This precludes *the Src family* from being considered a potential anaphor, since *Src* is a rigid designator. A similar condition applies to noun phrases which are followed by a prepositional phrase cued by *of*. For example, *the symptoms* in *the symptoms of lupus erythematosus* is ruled out as an anaphor.
- The noun phrase is cataphoric. We distinguish cataphoric phrases as those that contain the word *following*, as in *the following signs*.
- The number feature of the noun phrase head is incompatible with that of the determiner. For example, in the fragment *Both short-term dynamic psychotherapy and cognitive therapy have a place …*, *Both short-term dynamic psychotherapy* is chunked as an individual noun phrase and this constraint rules it out as a potential anaphor, since the determiner *both* is plural and the head *psychotherapy* is singular. This step is applied mainly to address a shortcoming of noun phrase chunking, even though the number agreement principle between the head and the determiner is general.
- The head of the noun phrase is not associated with a UMLS Metathesaurus concept. These are excluded due to lack of semantic information to use in subsequent steps.

#### Anaphor-antecedent linking

In the SemRep pipeline, anaphor-antecedent linking is performed before indicator rules and argument identification rules are applied to generate semantic predications, so that argument identification rules can take the results of anaphora resolution into account when determining the arguments of predicates.

As preparation for this phase, we combine the linguistic analyses from the sentences prior to the sentence containing the sortal anaphor under consideration, including coordination information. Anaphora resolution needs to take into consideration the entire discourse preceding the anaphor, especially in the context of MEDLINE abstracts, which are often relatively short.

The next step in anaphor-antecedent linking is selection of antecedents consonant with the sortal anaphor. To select these antecedents, we process the noun phrases (including coordinate noun phrases) that precede the sortal anaphor. Two consonance criteria are applied: *semantic consonance* and *number agreement*.

Semantic consonance is concerned with the semantic compatibility of the sortal anaphor and the candidate antecedent noun phrase and is defined in terms of *hypernymy*. We use the term hypernymy in a broader sense than previous work [4] where it was defined as a UMLS Metathesaurus-based hierarchical relationship. We consider a word or a multi-word expression *A* to be a hypernym of another, *B*, if one of the following holds:

- The UMLS concept corresponding to *A* is an ancestor of the UMLS concept corresponding to *B* AND they are not in a meronymic (part-whole) relationship (Taxonomy constraint)
- *B* belongs to a UMLS semantic group [54], which has as one of its associated headwords the headword of *A* (Headword constraint)
- *A* and *B* have the same headword but map to different UMLS Metathesaurus concepts and the number of tokens in *B* is greater than that in *A* (Shared Headword constraint)

The Taxonomy constraint is similar to the definition of hypernymy in Rindflesch and Fiszman [4]. Meronymy is assumed between *A* and *B* if their UMLS concepts both belong to Anatomy semantic group and they do not have *Cell* semantic type; in other words, if they both correspond to biological units higher than the cell. This constraint is necessary since the UMLS concept hierarchy encodes meronymic as well as taxonomic relationships. While meronymy may be useful for associative coreference [3], we did not find it useful for sortal anaphora. The Taxonomy constraint predicts semantic compatibility between *cetirizine* and *the drug*, while it finds that *right ventricle* and *heart* are incompatible, since the relationship between them is one of meronymy.

For the Headword constraint, we developed a headword list for several semantic groups based on our training set. For example, Disorder headwords include *condition*, *ailment*, *abnormality*, and *problem*, while the Therapeutic Modality headwords include *medication*, *intervention*, and *agent*. Such word lists are useful to compensate for the fact that UMLS concepts corresponding to such general terms are often not in the expected taxonomic relation with specific instances of these semantic classes. The Headword constraint predicts compatibility between *the illness* and *Immune reconstitution inflammatory syndrome*, which are not in a taxonomic relationship in the UMLS, for example. On the other hand, the Shared Headword constraint predicts compatibility between *the reaction* and *anaphylactoid reaction*, because *reaction* and *anaphylactoid reaction* are mapped to different UMLS Metathesaurus concepts and they share the same headword. Finally, we stipulate that neither the sortal anaphor nor the antecedent candidate belong to the semantic group Concept, which includes semantic types such as Idea or Concept, Conceptual Entity, and Functional Concept, and is too broad and heterogenous to be useful in anaphora resolution. For candidates that are coordinate noun phrases, the semantic consonance constraints are applied between the sortal anaphor and each of the conjuncts in the coordinate noun phrase.

The other consonance measure, number agreement, is a commonly used feature in coreference resolution. Our implementation uses the number feature provided by the SPECIALIST Lexicon. The sortal anaphor and an antecedent candidate are taken as compatible with respect to number if their heads agree on this feature (i.e., if both are plural or both are singular). The number feature for unknown words is taken as singular. Number agreement also takes into account coordinate noun phrases: a plural sortal anaphor is taken to be consonant with an antecedent candidate that is a coordinate noun phrase.

The anaphora resolution process is terminated if candidate antecedent selection results in no compatible antecedents or in a single compatible antecedent. In the former case, the anaphor may be a universal anaphor (e.g., *this study*), which can refer to clauses, full sentences, or even the full discourse; resolution of such phenomena is beyond the scope of this study. In the latter case, we simply take the only compatible antecedent as the true antecedent and generate an anaphora link between the anaphor and the antecedent. On the other hand, if there are multiple compatible antecedent candidates, we predict the best antecedent based on its salience. The following steps are taken to identify the most salient of the antecedent candidates:

1. If there are antecedent candidates in the same sentence, the one closest to the anaphor is taken as the antecedent.
2. Else, we move to the closest preceding sentence with compatible antecedent candidates and the leftmost compatible candidate in that sentence is chosen as the antecedent.

These steps seek to predict discourse salience of entities, in a sense similar to prediction of the *preferred center* in Centering Theory [18].

### Integrating anaphora resolution with relation generation

After generating anaphora links with the steps outlined above, SemRep attempts to use these links in relation generation, if appropriate. For an anaphora link to be used in relation generation, we require that the sortal anaphor noun phrase serve as the subject or object argument of a predicate. In such cases, rather than using the sortal anaphor in the predication, we simply substitute it with its antecedent(s) as the relevant argument(s). The rest of the relation generation procedure remains the same. In cases where sortal anaphor does not serve as an argument of a predicate, the corresponding anaphora link simply remains unused. For instance, in Example (1), the anaphor *these drugs* was recognized as the subject of the predicate *treatment*, which indicates a TREATS relation. With no anaphora resolution, relation generation would simply generate the predication Drugs-TREATS-PAH. With anaphora resolution, the subject argument in this predication (Drugs) is replaced by the UMLS concepts corresponding to the antecedents (Prostacyclin analogues, Endothelin receptor antagonists, and Phosphodiesterase type 5 inhibitors), resulting in three informative predications instead of the less informative Drugs-TREATS-PAH.

### Evaluation

In this study, we evaluated both anaphora resolution and its contribution to relation extraction. Additionally, we assessed the quantitative impact of anaphora resolution on the PubMed scale repository of biomedical relations supported by SemRep, SemMedDB [12].

As a baseline method for anaphora resolution, we considered a noun phrase containing one of the determiners or adjectives of interest (see *Anaphor detection* section) as a sortal anaphor and took the closest preceding noun phrase whose head word and number match those of the sortal anaphor as its antecedent. This baseline method is a more informed one than the one used by Segura-Bedmar et al. [44] and Kilicoglu and Demner-Fushman [55], who simply considered the closest preceding noun phrase as the antecedent.

To evaluate anaphora resolution, we used the test set portion of the annotated corpus and calculated precision, recall, and F_1_ score. In calculating these metrics, we used relaxed matching criteria, due to the fact that SemRep normalizes arguments of all relations (anaphora and others) from text mentions to UMLS Metathesaurus concepts. Three matching criteria are defined. An anaphora relation generated by SemRep is considered a true positive if:

- The character offsets of its arguments (anaphor and antecedent) overlap with those of a relation in the reference standard and the semantic types of the arguments match (i.e., approximate match).
- OR one or both of its arguments are subsumed by a span annotation in the reference standard (i.e., no explicit semantic type matching is required).
- OR the concept corresponding to the antecedent matches that of the antecedent in the relation in the reference standard (i.e., no antecedent character offset overlap is required).

To illustrate why the second criterion is necessary, consider the following example:

(3) An adult male bullmastiff dog* was treated for paraparesis and ataxia due to discospondylitis and disc herniation. At this time, *the dog* had a nonhealing ulcer between the pads of the left hindfoot.*

In this example, the first phrase *An adult male bullmastiff dog* was annotated as the antecedent (with generic SPAN type), which cannot be fully mapped to a UMLS concept. The algorithm identifies this noun phrase as the antecedent; however, it uses the concept corresponding to its head *dog* as the argument of the anaphora. Using the second evaluation criterion, we consider such cases true positives.

The third criterion is probably the most relevant evaluation criterion for SemRep, since SemRep is specifically concerned with this ontological semantic level. Consider the example below:

(4) *A radioaerosol technique was used to assess the effects on mucus clearance of 14 days treatment with *formoterol or tiotropium*, as well as single doses of these drugs. RESULTS: The 4 h whole lung retention of radioaerosol was significantly higher after 14 days treatment with *tiotropium (*P= 0.016), but not after 14 days treatment with *formoterol*. However, patients bronchodilated after 14 days treatment with *both drugs*, so that the deposited radioaerosol may have had an increased distance to travel in order to be cleared by mucociliary action.*

In this example, the anaphor *both drugs* refers to the drugs *tiotropium* and *formoterol*. Following annotation guidelines, the annotators annotated the closest mentions to the anaphor in the sentence preceding the one with the anaphor. The algorithm, on the other hand, identified as the antecedent the coordinate noun phrase *formoterol or tiotropium* in the first sentence. At the ontological semantic level, this is equivalent to the annotated antecedents, although the corresponding mentions do not overlap with those in the reference standard. Using the third criterion, we consider such cases true positives, as well.

For comparison with other anaphora resolution approaches and corpora, we also evaluated our approach against the BioNLP Protein Coreference Dataset [15], the most widely used coreference resolution corpus focusing on biomedical literature. We limited our evaluation on this dataset to sortal anaphora instances and did not consider the cases of pronominal anaphora. Sortal anaphora instances constitute 12.5 % of all the coreference relations in this corpus (n =69). These instances were identified by removing the anaphora relations indicated by pronominal anaphors from the dataset.

To assess the contribution of anaphora resolution to relation extraction, we processed with SemRep a set of 1 million MEDLINE citations that included abstracts, dated from April 2014 to June 2015. Two sets of output were generated: one set was generated without anaphora resolution and the other with anaphora resolution. We report results concerning the quantitative impact of anaphora resolution on this set. From the 1 million citation set, we also selected 300 sentences, for which anaphora resolution resulted in additional semantic predications. One of the authors (GR) manually examined the predications in these sets and evaluated their correctness. In the absence of a predication reference standard for these sentences, we only calculated precision. In this study, we recognize that categorizing predications as simply true positive or false positive does not adequately elucidate the contribution of anaphora resolution to relation extraction, because in some cases, anaphora resolution increases the specificity and informativeness of an existing predication, rather than generating a new additional predication. For instance, in Example (1), Drugs-TREATS-PHA is a somewhat uninformative (but not incorrect) predication and, if anaphora resolution succeeds, it would be substituted by the predication Prostacyclin analogues-TREATS-PAH, which is correct and more informative. To accommodate such positive changes, we marked predications like Drugs-TREATS-PHA as *partially correct* in this evaluation.

## Results and discussion

In this section, we present results pertaining to inter-annotator agreement as well as the evaluation results and discuss these results in detail. We conclude the section by providing an error analysis.

### Inter-annotator agreement

The results of inter-annotator agreement calculation are provided in Table 1.

**Table 1:** Inter-annotator agreement computed using F_1_ score

	Anaphoric mentions		Anaphora relations	
Batch	Exact	Approximate	Exact	Approximate
1	0.43	0.46	0.10	0.28
2	0.74	0.74	0.34	0.43
3	0.90	0.91	0.81	0.88

The inter-annotator agreement showed a clear improvement trend over three iterations, indicating that with sufficient guidelines and practice, good agreement can be achieved for this task. Discussing the annotation differences and reconciling them before moving on to the next batch also seem beneficial.

### Annotation statistics

The distribution of anaphoric mentions and anaphora relations in the double-annotated set and the single-annotated set is shown in Table 2. The average numbers of tokens, mentions, and relations are given in parentheses. We also show the number of additional entity annotations (SPAN) added to the corpus, corresponding to named entities missed by SemRep (and MetaMap) that were found to be relevant for anaphora resolution task.

**Table 2:** Annotation statistics

	Tokens	span annotations	Anaphoric mentions	Anaphora relations
Training (149)	42,822 (287.4)	211 (1.42)	379 (2.54)	427 (2.87)
Test (171)	50,458 (295.1)	265 (1.55)	564 (3.30)	754 (4.41)
TOTAL (320)	93,280 (291.5)	476 (1.49)	943 (2.95)	1181 (3.69)

The average numbers of anaphoric mentions and relations are higher in the test set than in the training set. In creating the training set, we did not filter citations based on the presence of anaphoric expressions (e.g., *the gene*). We did, however, perform this filtering step in the test set, which led to a higher proportion of citations with anaphora relations (14.6 % in the training set vs. 21.5 % in the test set).

It is noteworthy that there are about 25 % more anaphora relations than anaphoric mentions, providing further evidence regarding the prevalence of set-membership relations in biomedical corpora [3]. The set-membership relations involved up to 9 members. The distribution of member counts for set-membership relations is given in Table 3. The average number of antecedents for an anaphor indicating set-membership anaphora is 2.66.

**Table 3:** Member counts in set-membership relations

Member count	Set-membership relations	%
2	166	60.4
3	66	24.0
4	29	10.5
5	6	2.1
6	3	1.1
7	3	1.1
8	1	0.4
9	1	0.4
TOTAL	275	100.0

It should also be noted that more than one SPAN annotation was created per citation (1.49 on average), which indicates that the antecedents often involve entities that do not map to UMLS concepts in a straightforward manner (such as *An adult male bullmastiff dog* discussed above). We also found that approximately 85 % of all anaphora relations were inter-sentential, showing that coreference resolution is highly important for discourse-level text understanding.

### Anaphora resolution

The evaluation results of anaphora relations are given in Table 4. The baseline method, perhaps unsurprisingly, performs poorly. It yields better precision than recall, indicating that while simple head word match is a simple and useful criterion for detecting anaphora, it needs to be augmented with other semantic constraints for reasonable performance. Anaphora resolution in SemRep provides a 4-fold increase in F_1_ score compared to the baseline method. Interestingly, while SemRep favors precision over recall in relation extraction, precision and recall figures for the anaphora resolution algorithm are relatively close.

**Table 4:** Anaphora resolution evaluation

System	Precision	Recall	F_1_ score
Baseline	35.2	7.1	11.9
Anaphora resolution algorithm	64.6	55.2	59.6

To measure the effect of various anaphora resolution components on the overall performance, we also performed an ablation study in which we removed these components and recalculated evaluation metrics. The results of this study are provided in Table 5.

**Table 5:** Ablation study results

Removed component	Precision	Recall	F_1_ score
Anaphoricity filter	53.4	58.1	55.6
Taxonomy constraint	55.1	13.6	21.8
Headword constraint	65.5	52.1	58.0
Shared Headword constraint	64.7	54.5	59.1
Number constraint	57.7	50.8	54.0
Set-membership processing	46.6	21.6	29.5

The anaphoricity filter improved performance significantly (4 percentage points), in contrast to previous studies in which such filtering often resulted in poorer results [21]. Among the semantic compatibility measures, the Taxonomy constraint had the greatest impact on performance (an improvement of more than 37 points). The effects of the Headword and Shared Headword constraints were much smaller (1.6 and 0.5 points respectively). The Number agreement yielded a noticeable, positive improvement (about 5.5 points). These results show that a strong semantic constraint coupled with number agreement can successfully identify antecedent candidates. The effect of removing the set-membership recognition component was close to that of removing the Taxonomy constraint, lowering the F_1_ score to half, another indication of the importance of set-membership anaphora in biomedical literature.

Comparing intra-sentential to inter-sentential resolution results revealed the interesting phenomenon that the system, perhaps counter-intuitively, performs better on anaphora relations crossing sentence boundaries (shown in Table 6). Analyzing the results, we noted that the majority of intra-sentential antecedents are in structures of coordination, and that the lower performance of the system on sentence-bound anaphora relations can be partly attributed to the difficulty of resolving coordination.

**Table 6:** Performance on intra- vs. inter-sentential anaphora

Processing	Precision	Recall	F_1_ score
Intra-sentential	55.7	43.3	48.8
Inter-sentential	64.5	52.2	57.7

Our evaluation on sortal anaphora relations in the development portion of the BioNLP Protein Coreference Dataset yielded the results given in Table 7. This dataset incorporates both sortal and pronominal anaphora relations, and not all studies conducted on this dataset have reported sortal anaphora resolution performance separately. Among those that did, the best results were reported by D’Souza and Ng [35], who also reported the best overall performance. On this dataset, our methodology performs slightly better than theirs with respect to F_1_ score, with higher recall than theirs at the expense of lower precision. Our low precision is mostly due to the fact that we did not provide gold protein entities as input to the system and did not limit ourselves to resolution of protein/gene-related anaphora only. It is reasonable to assume that we could increase precision on this dataset by adding a few, simple post-processing rules.

**Table 7:** Anaphora resolution evaluation on the BioNLP protein coreference dataset (development portion)

System	Precision	Recall	F_1_ score
D’Souza and Ng [35]	58.3	6.9	12.4
Our approach	11.5	14.5	12.8

#### Effect on semantic interpretation

Processing 300 sentences from MEDLINE with SemRep with and without the anaphora resolution option, we found that there was an increase of approximately 2 % in the number of predications solely due to anaphora resolution (from 1737 predications to 1771 predications). The increase may seem minor; however, this number does not fully capture the effect of anaphora resolution. We found that 1471 predications remained unchanged with and without anaphora resolution, indicating that 15.3 % of the predications generated without anaphora resolution (266 predications) were changed to some extent with anaphora resolution. We analyzed the changes with respect to these 266 predications to assess whether they were positive changes or not. The results, shown in Table 8, indicate that the effect of sortal anaphora resolution is positive overall; half of the changes (50 %) involve uninformative predications being replaced by more specific and informative ones (partially correct → true positive).

**Table 8:** Effect of anaphora resolution on semantic interpretation

Change	Count	%
Partially correct → True positive	150	50
False positive → False positive	102	34
Partially correct → False positive	42	14
True positive → False positive	4	1.4
Partially correct → Partially correct	1	0.3
True positive → True positive	1	0.3

In 34 % of the cases, anaphora resolution may have generated a correct relation; however, this did not lead to improvement because the original predication was a precision error in the first place (false positive → false positive). Consider the example below:

(5)
  *The cyst fluids were shown to be a rich source for *acidic glycoproteins*. The study of *these proteins* can potentially lead to the identification of more effective *biomarkers* for *ovarian cancer.
  ^∗^Proteins-PREDISPOSES-Ovarian Carcinoma^1^
  Glycoproteins-PREDISPOSES-Ovarian Carcinoma

Although the anaphora relation between *these proteins* and *acidic glycoproteins* is captured correctly by the algorithm, the resulting predication in Example (5c) is a precision error, because the original predication that it is based on (Example (5b)) is incorrect, due to misidentification of the subject as *proteins*, instead of the correct subject *biomarkers*.

Negative changes constitute 15.4 % of the changes (partially correct → false positive; true positive → false positive). An example of such a negative change is given below:

(6)
  NASH* is a distinct entity from NAFLD, and is characterized by the presence of *inflammation* with hepatocytes damage, with or without fibrosis. While several therapeutic strategies have been proposed to improve this condition, the present review aims to discuss nonmedicinal *interventions* used to reduce liver involvement or to *preventthe disease* altogether.*
  Interventions-PREVENTS-Disease
  ^∗^Interventions-PREVENTS-Inflammation

Without anaphora resolution, SemRep generates the predication in Example (6b), which, while correct, is uninformative, and therefore, considered partially correct. Because the acronym *NASH* cannot be resolved to *nonalcoholic steatohepatitis*, anaphora resolution algorithm does not recognize it as an antecedent candidate, identifying *inflammation* instead as the antecedent for the anaphora *the disease*. This leads to the incorrect predication in Example (6c).

Our analysis also revealed that set-membership anaphora relations can amplify both the positive and negative impact of anaphora resolution on semantic interpretation. Example (1) illustrates the amplification of positive impact, in which one uninformative predication is replaced with three informative ones thanks to a single anaphora relation. Example (7a) shows an instance where the effect is negative, with one partially correct (uninformative) predication being replaced by four incorrect ones, due to misidentification of antecedents. The antecedent for the anaphor *The genes* was found to be *Bad, Bid, Fas, and TNF*, instead of *API5, AIFM1, and NFkappaB1*. However, note that the negative effect could have been even more pronounced if the terms coordinated with *Bad, Bid, Fas, and TNF* (i.e., *the caspase family* and *Bcl-2*) satisfied the semantic consonance constraints, which would lead to two additional precision errors.

(7)
  *Meanwhile, three genes (*API5, AIFM1, and NFkappaB1*) showed changes of expression in the hippocampus of Ts65Dn mice compared with normal mice…However, some well-known genes related to cell apoptosis, such as the caspase family, Bcl-2, *Bad, Bid, Fas, and TNF*, did not show changes in expression levels. *The genes* we found which were differentially *expressed* in the *hippocampus* of Ts65Dn mice may be closely related to cell apoptosis.*
  Genes-PART_OF-Entire hippocampus
  ^∗^BAD gene-PART_OF-Entire hippocampus
    ^∗^TNF gene-PART_OF-Entire hippocampus
    ^∗^FAS gene-PART_OF-Entire hippocampus
    ^∗^BID gene-PART_OF-Entire hippocampus

Finally, we compared overall SemRep performance with and without anaphora resolution. In previous work, we developed a reference standard dataset for SemRep benchmarking [49]; however, predications were annotated at the sentence level, ignoring anaphora completely. Therefore, it is not suitable for assessing overall SemRep performance in the context of the current study. Instead, we performed a post hoc evaluation of predications generated by SemRep with and without anaphora resolution on 300 sentences, calculating precision only. Calculating recall, and by the same token F_1_ score, would require a non-trivial, labor-intensive annotation study, especially considering that it would have to involve document-level conceptual annotation. Precision calculation is complicated by *partially correct* predications that we referred to earlier. If they are considered true positives, the impact of anaphora resolution may not be evident at all. On the other hand, if they are considered false positives, the impact of anaphora resolution may be overestimated. Considering this complexity, we report precision in two ways: a) excluding partially correct predications from calculation, b) awarding them 0.5 points. The results, given in Table 9, clearly show the positive impact of anaphora resolution on the precision of SemRep predications; in both calculations, precision is improved (by 1.3 and 2.1 points, respectively). Given that anaphora resolution is mainly a recall improvement strategy for relation extraction, this indicates that overall F_1_ score is improved, as well.

**Table 9:** Overall SemRep precision with and without anaphora resolution

System	Precision
*Partially correct predications ignored*	
Base SemRep	58.0
Enhanced with anaphora resolution	59.3
*Partially correct predications awarded 0.5 points*	
Base SemRep	57.1
Enhanced with anaphora resolution	59.2

#### Effect of sortal anaphora resolution at PubMed scale

The final evaluation concerned the effect of anaphora resolution on SemRep results at PubMed scale. To assess this effect, we considered anaphora relations and predications extracted from a randomly selected set of 1 million abstracts (corresponding to about 4 % of the entire MEDLINE corpus). We found that our algorithm extracted a total of 504,604 anaphora relations from this set of abstracts, indicating an average of about 0.5 anaphora relation per abstract. We also found that the number of predications increased from 5,187,549 to 5,197,458 including duplicates, an increase of approximately 0.2 %. This is about one-tenth of the increase in the set of 300 MEDLINE sentences we discussed above. However, this comparatively small increase is not surprising; for that set, we specifically analyzed sentences in which anaphora resolution led to changes in semantic interpretation. Extrapolating from the increase in the overall number of predications due to anaphora resolution in that smaller set versus the larger 1 million abstract set (2 % vs. 0.2 %) and the ratio of predications affected by anaphora resolution on that smaller set (15 %), we can estimate that anaphora resolution triggers a change in about 1.5 % of the predications in the larger set of 1 million abstracts. Considering that our PubMed scale database of semantic predications, SemMedDB [12], contains more than 82 million predications in its latest release (through June 30th, 2015), these seemingly small increases are still likely to constitute a significant enhancement in the amount of knowledge mined from the literature and the specificity of that knowledge. Finally, we analyzed the proportional change in the number of predicate types. We found that 91 % of the predicate types had an increased number of relations after anaphora resolution. Using paired sample t-test, we found that changes in the number of predications per predicate type were statistically significant (*p* < 0.0001). The rates of change for the top 10 most common predicate types are provided in Table 10. The largest increase involved predicates concerning molecular level relations, such as gene-gene and gene-drug relations. The highest increase was in STIMULATES and INHIBITS relations (0.46 %) followed by ASSOCIATED_WITH (0.45 %), a gene-disease relation. On the other hand, several minor clinical relations exhibited fewer relations (PRECEDES had a drop of 0.82 % and MANIFESTATION_OF a drop of 0.56 %). This finding illustrates the prevalence of anaphora relations in molecular biology literature and highlights the importance of resolving them for biological curation tasks.

**Table 10:** The rates of change for the top 10 predicate types in 1 million Medline abstracts

Predicate type	Change (%)
PROCESS_OF	+0.09
LOCATION_OF	+0.20
TREATS	+0.17
PART_OF	+0.19
ISA	+0.12
AFFECTS	+0.33
USES	+0.15
COEXISTS_WITH	+0.26
INTERACTS_WITH	+0.44
ASSOCIATED_WITH	+0.45

### Error analysis

**Fig. 3:**
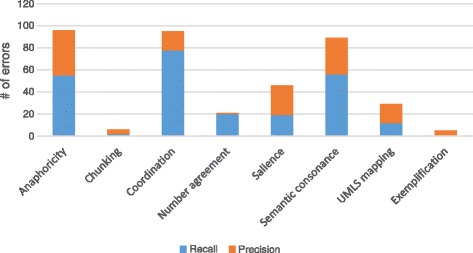
Distribution of error types. Distribution of precision and recall errors

We analyzed the errors made by the anaphora resolution component of SemRep, categorizing them by the underlying cause of the error. The distribution of error types is illustrated in Fig. 3. Note that an error can be due to multiple causes; therefore, the total number of errors in Fig. 3 exceeds the total number of actual precision/recall error instances.

Three major error types were caused by coordination processing, anaphoricity filtering and semantic consonance measures. While coordination processing errors predominantly led to recall errors (82 %), precision vs. recall errors due to anaphoricity filtering and semantic constraints were more evenly distributed (57 % and 63 % are recall errors, respectively). Among the less prevalent error types, number constraint errors were almost exclusively recall errors (95 %), while salience rules and UMLS mappings caused a higher number of precision errors (both 59 %).

Anaphoricity filtering errors constituted 25 % of all errors. Some of these errors had to do with misidentification of *rigid designators*, leading the system to ignore legitimate anaphors. In the following example, the underlined phrase was not considered an anaphor, since its modifier *PAH* is taken as a rigid designator, because it is mapped to a UMLS Metathesaurus concept.

(8) *Therefore, data that support the long-term therapeutic benefits of *these long-termPAH therapies* are limited and derived primarily from uncontrolled, observational studies …*

We also found precision errors due to the shortcomings of the anaphoricity filter. In the following example, the appositive construction between *sildenafil* and *the first phosphodiesterase inhibitor* was not recognized. The algorithm, therefore, considered the latter a sortal anaphor and linked it to the phrase *Revatio*.

(9) *this paper …provides an overview of clinically available phosphodiesterase inhibitors and discusses tadalafil in relationship to sildenafil (*Revatio(R)), thefirst phosphodiesterase inhibitor* approved for PAH.*

As discussed above, removing the anaphoricity filter lowered the F_1_ score by 4 %, lowering precision while increasing recall, showing that its overall effect was positive, although a more nuanced constraint could further improve results.

Despite the fact that coordination processing benefitted anaphora resolution significantly by addressing set-membership anaphora, as shown in Table 5, still a significant number of errors were caused by issues in coordination processing (25 % of all errors). In the following example, the serial coordination between *oxidative stress*, *inflammation*, and *impaired Nrf2 activation* was not identified by SemRep’s coordination processing, because *impaired Nrf2 activation* was not mapped to a UMLS concept in the Disorder semantic group. This led the algorithm to miss three anaphora relations, between *These abnormalities* and the underlined coordinate noun phrases.

(10) *…progressive focal glomerulosclerosis in the Imai rats is associated with *oxidative stress, inflammation*, and *impaired Nrf2 activation. These abnormalities* are accompanied by activation of …*

In addition to recall errors such as the aforementioned, coordination processing may also trigger precision errors. In the example that follows, *insulin glargine* was erroneously found to be coordinated with *nateglinide* and *acarbose*, which led to three anaphora relations, instead of two correct relations identified by the system (between *Both drugs* and the antecedents *nateglinide* and *acarbose*). This error could also be prevented by stipulating that an anaphor involving the determiner *both* can only have two antecedents. The incremental nature of SemRep allows implementing such a rule easily.

(11) *After fasting glucose was optimized by *insulin glargine, nateglinide or acarbose* was initiated and then crossed over after second wash out period. …*Both drugs …

Coordination resolution in SemRep is particularly poor in identifying serial coordination; enhancements made in processing this linguistic phenomenon can have a positive effect on anaphora resolution as well. However, coordination processing is likely to remain one of the main challenges with anaphora resolution, since coordinate constructions can get very complex in biomedical text. In the example below, seven anaphora relations were missed, since the appropriate coordination structure cannot be recognized due to the intervening percentage values.

(12) *The percentage of patients discontinued treatment within 12 months was 41.4 % for *chlorpromazine, 39.5 % for sulpiride, 36.7 % for clozapine, 40.2 % for risperidone, 39.6 % for olanzapine, 46.9 % for quetiapine, and 40.2 % for aripiprazole*, a nonsignificant difference (p=0.717); there were no significant differences among *these seven treatments* on discontinuation due to relapse, …*

Errors due to one of the semantic consonance measures led to 23 % of all errors. In the following example, the anaphor *The chemotherapy regimen* and the antecedent *adjuvant chemotherapy* are not found to be semantically consonant, since there is no taxonomic relation between the corresponding concepts in the UMLS, and their headwords do not match.

(13) *From January 1993 to March 1998, 268 patients were randomized to *adjuvant chemotherapy* (135 patients) or surgery alone (133 patients). All patients underwent gastrectomy with D2 or greater lymph node dissection. *The chemotherapy regimen*consisted …*

On the other hand, lack of a full UMLS mapping for *impaired Nrf2 activation* in Example (10) above led to an error in coordination processing, which also relies on such information, and it resulted in a negative effect on anaphora resolution.

Number agreement caused some anaphor-antecedent linking errors, although not many (5 % of all errors). In the following example, the anaphor *the drug* and the antecedent *neuroleptics* do not agree in number, leading to a recall error.

(14) *Stereotypies and orobuccolingual dyskinesias are the most frequently observed tardive disorders, particularly in the elderly population exposed to *neuroleptics*, …. The development of these disorders is dependent on the potency of *the drug*, duration of exposure, and …*

Shortcomings of the salience-based best antecedent selection also triggered anaphor-antecedent linking errors (12 %). Consider the example below:

(15) *We report a case of pulmonary infection caused by a rare *Nocardia species, Nocardia beijingensis*, in a 48-year-old man who received multiple immunosuppressive therapy after renal transplantation. *This pathogen* was isolated from a bronchoscopic protected specimen brush ….*

In this example, the salience-based selection prefers *Nocardia species* over *Nocardia beijingensis* as antecedent because it is the leftmost compatible antecedent candidate in the sentence. This leads to both a precision and a recall error.

A small number of precision errors (1 % of all errors) were due to exemplification instances, which annotators were instructed not to annotate. In the following example, the antecedent for the anaphor *these drugs* was annotated as *ion channel modulators*. The algorithm, on the other hand, identifies *pregabalin, gabapentin,* and *carbamazepine* as the antecedents, leading to three precision errors.

(16) *Among the substances which are commonly used are *ion channel modulators* (e.g. pregabalin, gabapentin, carbamazepine). The aim of this study was to investigate the use of *these drugs* in clinical practice in a larger patient cohort.*

These errors can be considered soft errors, since the extracted anaphora relations can still be useful for the downstream relation generation step.

## Conclusions

We presented a general, linguistically-oriented methodology that relies heavily on UMLS semantic knowledge to recognize sortal anaphora relations in biomedical literature. In contrast to previous studies on this topic, we did not focus on specific entity types. Our semantic approach resulted in a 4-fold increase in F_1_ score over the baseline. The methodology has been incorporated into a general biomedical semantic relation extraction tool, SemRep, and we showed that its overall effect on relation extraction is positive. Since SemRep supports a literature-based biomedical knowledge management tool, Semantic Medline [11], and a PubMed-scale repository of semantic relations, SemMedDB [12], which in turn support tasks such as literature-based discovery [56] and question answering [57], we believe that enhancing SemRep with anaphora resolution will benefit such downstream applications. While our study focused on MEDLINE citations, the methodology makes few assumptions regarding the type of input text. Therefore, we believe it would be largely applicable to full-text articles, although discourse-based constraints (e.g., the distance between the anaphor and the antecedent candidate) would probably need to be taken into account. With relatively short length of MEDLINE citations, such constraints were not needed. Anaphora resolution is made available as an option in the web-based SemRep tool^2^. The annotated corpus used for training and evaluation is publicly available at http://skr3.nlm.nih.gov/SortalAnaphora. A UMLS license is required.

While the overall effect of anaphora resolution is positive and renders more informative predications, the evaluation and error analysis revealed areas for potential improvement. These include a more nuanced approach to salience-based best antecedent selection for intra-sentential anaphora relations, an improved method to detect rigid designators, and an improved coordination processing, which would enhance not only anaphora resolution but also the core semantic interpretation capability of SemRep. Future work also involves pronominal anaphora resolution, which we have not attempted in this study due to its relative sparsity in biomedical literature.

## Endnotes

^1^ The asterisk (^∗^) indicates an incorrect predication.

^2^http://ii.nlm.nih.gov/Interactive/UTS_Required/semrep.shtml

## Additional file

Additional file 1 Sortal anaphora annotation guidelines. The sortal anaphora annotation guidelines provided to the annotators and refined/expanded in several iterations. (PDF 79.1 kb)
