# Supplementary material for: Sortal anaphora resolution to enhance relation extraction from biomedical literature
Source: BMC Bioinformatics. 2016 Apr 14;17:163. doi: 10.1186/s12859-016-1009-6 (PMC4832532; doi:10.1186/s12859-016-1009-6)
Supplement: Additional file 1 — Sortal anaphora annotation guidelines. The sortal anaphora annotation guidelines provided to the annotators and refined/expanded in several iterations. (PDF 79.1 kb) [file 12859_2016_1009_MOESM1_ESM.pdf]

Sortal Anaphora Annotation:

The goal of this study is to annotate Medline citations with sortal anaphora relations. This annotation will be beneficial in evaluating the sortal anaphora resolution extension to SemRep.

Anaphoric relations are grammatical constructions whereby a noun phrase mentioned earlier in a sentence or abstract is later referenced inexplicitly, without restating the actual noun phrase but instead replacing it with a general term that is representative of it. Examples of this include instances such as “aspirin” being later referenced as “this drug”, and “cancer” being later referenced as “the disease”, as well as pronominal phrases like “it”, “he”, and “she” being used to reference back to “the table”, “Joe”, or “the doctor”. In these structures, the noun phrase being referred to is called the *antecedent*, and the referential noun phrase is called the *anaphor*. Sortal anaphors are the subset of anaphors that do not include pronominal anaphors such as “it”, “he”, “she”, etc., but include definite, demonstrative or distributive noun phrases, such as ones that start with the determiners “the”, “this”, “these”, “those”, “their”, “that”, “both”, “either”, “such”, etc. A sortal anaphora example is given below. The antecedent is in red and the anaphor has yellow background:

(1) It has become clear that cancer is not merely a growth of autonomously proliferating cells, but that other non-malignant cell types are a functional part of the disease.

We use web-based *brat* annotation tool for coreference annotation. The URL for this study is http://******. Each annotator is assigned a separate folder under that URL (e.g., ******). Each folder contains all Medline citations to be annotated as separate documents.

Each Medline citation has been pre-annotated with entities and relations extracted by SemRep in order to ease the annotation task. Each entity annotation is linked to the source it comes from (UMLS or EntrezGene). These automatic annotations, while not necessarily correct, are intended to provide some context for the task.

The annotation steps are described below, with the following sentence as the running example (from PMID 2109831).

(2) Sex determination compared in Drosophila and Caenorhabditis. Fruitflies and nematodes show many similarities in the general organization of the gene networks that control sexual dimorphism and dosage compensation. In contrast, the underlying molecular mechanisms appear to be very different in these two species.

I. Annotate the anaphor with the entity type SortalAnaphor. In this case, annotate “these two species”. Make sure to only annotate the full extent of the anaphor and nothing more. For instance, in the example above, the determiner “these” qualifies the expression as anaphor and, thus, should be annotated. On the other hand, the preposition “in”, which appears before the anaphor, should not be part of the anaphor annotation.

II. Identify the antecedent(s) and annotate it unless it already is pre-annoatated. In the example, there are two antecedents “Fruitflies” and “nematodes”, both of which are pre-annotated. If the antecedent is not pre-annotated, simply create an entity with the type SPAN.

III. Create a Coreference relation between the anaphor and the antecedent. In this example, Expression annotation associated with “these two species” should be linked to “Fruitflies” and “nematodes”. If there are multiple antecedent candidates (for example, in the first sentence above, “Drosophila” co-refers with “these two species” as well), pick the one closest to the anaphor (i.e., “Fruitflies”).

   a. If an anaphor refers to multiple antecedents, create a separate annotation between the anaphor and each antecedent. That is, for the sentence above, create two Coreference annotations:

      i. These two species -> Fruitflies

      ii. These two species -> nematodes

Notes:

- All instances of sortal anaphora should be annotated, regardless of whether they can be linked to a term or not (e.g., `these findings, the inhibition, this process`).

- Hypernymic (e.g., appositive) relations should not be annotated.

  - `This may occur through the ability of IL-10 to induce expression of ~~the gene~~, ~~suppressor of cytokine signaling 3 (SOCS3)~~`

- Cataphoric relations should not be annotated.

- Coreferential relations that do not involve sortal anaphora (e.g., pronominal anaphora or non-anaphoric coreference) should not be annotated.

  - Pronominal: `Our results suggest that the ~~JNK~~ pathway may regulate NF-kappa B-mediated gene transcription through ~~its~~ phosphorylation`

  - Non-anaphoric: `~~The ERK pathway-specific inhibitor PD98059~~ inhibited IL-6 secretion from monocytes… By using ~~PD98059~~, we demonstrated that the Raf1/MEK1/ERK1/2 pathway`

- In general, a SortalAnaphor instance should not be annotated as the antecedent. In these cases, try to identify the actual specific term that the expression refers to.

  - `We have used ~~this DNA fragment~~ to isolate and purify a 60-kDa protein binding to this fragment`

- Do not annotate exemplifications as antecedents (for exemplifications, look for clues like ‘e.g’, ‘including’, ‘such as’, etc.)

- The Th2-type cytokines, interleukin-4 (IL-4) and interleukin-13 (IL-13), induce expression of a distinct subset of genes in human monocytes, including FcepsilonRIIb (~~CD23~~), 15-lipoxygenase, IL-1 receptor antagonist (~~IL-1ra~~), and type I and type II IL-1 receptors (~~IL-1R~~). Type I interferons (IFN-alpha and IFN-beta) and type II interferon (IFN-gamma) inhibit induction of these genes by IL-4 and IL-13.

On the other hand, the following annotations are valid, due to the absence of exemplification.

- Comparison of KG1 to the PMA-unresponsive subline KG1a reveals differences in expression of TNF receptors 1 and 2; PKC isoforms alpha, beta I, beta II, and mu; and RelB, suggesting that these components/pathways are important for DC differentiation

- In the cases in which the antecedent involves relativizing modification, it is sufficient to annotate the head of the relative clause as the antecedent.

    - We monitored the fate of XY germ cells ~~placed in a female environment throughout development~~. Here we report that such germ cells respond to both cell-autonomous and somatic sex-determining signals, depending on the developmental stage.

- Acronyms can be annotated as antecedents.

Examples:

Background: Congestive heart failure (CHF) is a complex clinical syndrome with autonomic dysbalance and increased plasma levels of inflammatory cytokines, which further worsen the syndrome.

Findings of increased Pittsburgh Compound B (PiB) binding in subjects with Lewy Body Disease (LBD) compared with Parkinson's disease and dementia (PDD) may explain phenotype differences in the spectrum of Dementia with Lewy Bodies (DLB), and show promise in guiding future therapeutic trials aimed at this disease.

In this study, we evaluated the susceptibility of Escherichia coli, Pseudomonas aeruginosa and Salmonella typhi as well as spheroplasts of these bacteria to the compound, determining percent inhibition by turbidimetry.

The 8p11 myeloproliferative syndrome, also known as stem cell leukemia/lymphoma, is a rare, atypical, myeloproliferative disorder and lymphoid malignancy associated with chromosomal abnormalities involving the 8p11 chromosomal band. Translocations associated with this syndrome result in the fusion of the fibroblast growth factor receptor 1 (FGFR 1) gene with various partners, resulting in ligand-independent FGFR activity.

Recent reports have identified a similar clinical presentation beyond infancy called late-onset central hypoventilation syndrome (LO-CHS) as a disease

continuum of CCHS with similar and overlapping pathophysiology. However, some have proposed that the syndrome accompanied by hypothalamic dysfunction (HD) be classified as a distinct clinical entity, LO-CHS/HD.

CONCLUSION: Lemierre syndrome can result in infection spreading to the CNS, including cerebral subdural and epidural empyemas. This disease entity should be included in the differential diagnoses of CNS bacterial infections.

A common treatment for pediatric femur fractures is intramedullary nail (IMN) insertion. Elastic stable intramedullary nails (ESINs) are often used for these procedures in heavier patients, but the potential for complications and malunion is greater.

Liver resection offers the chance of a cure for liver cancer. However, when extended hepatectomies were performed in combination with resection of the inferior vena cava (IVC), the procedures were reported to have a surgical mortality rate in excess of 5 %. While most of these operations were performed with the use of veno-venous bypass, this study presents our experience performing the procedure without the bypass.

Exercise-induced intravascular haemolysis and "sport anemia" are widely reported in human sports medicine. It has been recognized also in horses, however, the clinical importance and the onset of this condition seem different than in human.

The high survival rate obtained in several studies supports the hypothesis that implants may be successfully osseointegrated when placed immediately after extraction of teeth presenting endodontic and periodontal lesions, provided that appropriate clinical procedures are performed before the implant surgical procedure such as meticulous cleaning, socket curettage/debridement, and chlorhexidine 0.12% rinse. However, more randomized controlled clinical trials with a longer follow-up are required to confirm this procedure as a safe treatment. Moreover, the outcome measures were not related to the type of infection; the classification of infection was often vague and varied among the studies.

Churg-Strauss syndrome is a vasculitis of medium to small sized vessels. Diagnosis is mainly clinical with findings of asthma, eosinophilia, rhinosinusitis and signs of vasculitis in major organs. CASE PRESENTATION: We present a case of a 19-year-old Persian male who developed signs and symptoms of this syndrome related to hyposensitization treatments for allergy control.

Although arthroscopic anchor suturing is commonly used for rotator cuff repair and achieves good results, certain shortcomings remain, including difficulty with reoperation in cases of retear, anchor dislodgement, knot impingement, and financial cost. In 2005, we developed an anchorless technique for arthroscopic transosseous suture rotator cuff repair. DESCRIPTION OF TECHNIQUE: After acromioplasty and adequate footprint decortication, three K-wires with perforated tips are inserted through the inferior margin of the greater tuberosity into the medial edge of the footprint using a customized aiming guide. After pulling the rotator cuff

stump laterally with a grasper, three K-wires are threaded through the rotator cuff and skin. Thereafter, five Number 2 polyester sutures are passed through three bone tunnels using the perforated tips of the K-wires. The surgery is completed by inserting two pairs of mattress sutures and three bridging sutures. METHODS: We investigated the retear rate (based on MR images at least 1 year after the procedure), total score on the UCLA Shoulder Rating Scale, axillary nerve preservation, and issues concerning bone tunnels with this technique in 384 shoulders in 380 patients (174 women [175 shoulders] and 206 men [209 shoulders]).

BACKGROUND: We analyzed the association of previous course-of-illness and other variables of clinical interest with a high frequency of both depressive or (hypo)manic episodes controlling for the effect of socio-demographic characteristics. METHODS: A total of 108 outpatients with a DSM-IV diagnosis of bipolar disorder (BD) were recruited. A retrospective and naturalistic study was conducted to examine the number of affective episodes and their relationship with socio-demographic, clinical and course-of-illness variables, including adherence to medication, type of medication used and the use of addictive substances. The episode frequency was estimated as the number of "major instances" of depression, hypomania and mania during the illness.

Neuroinflammation is a constant event in Alzheimer's disease (AD), but the current knowledge is insufficient to state whether inflammation is a cause, a promoter, or simply a secondary phenomenon in this inexorably progressive ailment. In the current paper, we review research data showing that inflammation is not a prerequisite for onset of dementia, and, although it may worsen the course of the disease, recent evidence shows that chronic inhibition of inflammatory pathways is not necessarily beneficial for patients.
